# Power struggles and organizational learning in crisis: the impact of power games on healthcare management during COVID-19

**DOI:** 10.1108/JHOM-08-2025-0495

**Published:** 2025-11-28

**Authors:** Ritva Gisela Rosenbäck, Ann Svensson

**Affiliations:** Department of Engineering Science, University West, Trollhättan, Sweden; School of Business, Economics and IT, University West, Trollhättan, Sweden

**Keywords:** Organizational learning, Crisis management, Power games, Sensegiving, Healthcare leadership, Resilience, COVID-19

## Abstract

**Purpose:**

This study aims to explore the impact of power games and leadership dynamics on organizational learning and resilience within healthcare organizations during the COVID-19 pandemic. By examining power struggles and sensegiving practices across hierarchical levels in two Swedish hospitals, the study investigates how these dynamics affected decision-making, crisis management and adaptive responses in a high-pressure environment.

**Design/methodology/approach:**

This qualitative study uses semi-structured interviews with key healthcare managers and personnel responsible for crisis management. A deductive analysis is applied using theoretical frameworks on organizational learning, power games and sensegiving. Three critical events are analyzed to illustrate the effects of power dynamics and leadership practices on learning and organizational adaptation.

**Findings:**

The study finds that power games and unilateral sensegiving significantly disrupted communication, decision-making and organizational learning. Informal leadership structures emerged to address immediate challenges, yet tensions with formal management highlighted the need to integrate these emergent practices into formal structures.

**Practical implications:**

This study highlights that effective pandemic crisis management in healthcare requires balancing formal authority with inclusive and adaptive practices. Key implications include grounding sensegiving in frontline input, adopting flexible “plan-to-plan” approaches, involving hospital-level managers in decision-making, prioritizing practical over political solutions and fostering service-oriented leadership that enables rather than controls.

**Originality/value:**

This study contributes to understanding how leadership and power dynamics shape learning and resilience during healthcare crises. By combining the insights with organizational learning and the Plan-Do-Study-Act cycle, it provides a novel perspective on integrating formal and informal leadership to enhance crisis management.

## 1. Introduction

Organizational change during crises is characterized by uncertainty, rapid decision-making, and complex power dynamics. In such contexts, the flow of information, along with the prior knowledge and interests of different groups, plays a crucial role in shaping how practical initiatives and learning emerge (Boin *et al*., 2016; Rosenbäck and Svensson, 2024; Svensson *et al*., 2025; Weick, 1995). Differences in the interpretation of information can lead to power struggles between groups within the organization, further complicating the crisis management process (Drori and Ellis, 2011).

Organizational changes in complex environments, such as healthcare, require continuous learning and adaptation (Deming, 1986; Gherardi and Nicolini, 1998; Weick, 1995). However, this process is rarely linear or entirely positive. While shared meaning-making and collective reflection can facilitate learning (Bruner, 1986; Gioia and Chittipeddi, 1991; Weick *et al*., 2005), they can also be disrupted by conflicting interests, power games, and fear (Courpasson, 2000; Drori and Ellis, 2011; Reissner, 2006).

For instance, the prolonged and complex nature of the COVID-19 pandemic forced healthcare organizations to adapt rapidly to shifting demands, high uncertainty, and emotional strain (Hølge-Hazelton *et al*., 2021; Leite *et al*., 2020; Rosenbäck and Svensson, 2023). Continuous improvement and learning were essential throughout the pandemic (Rosenbäck and Svensson, 2023; Sinha and Ola, 2021). Moreover, Rosenbäck and Svensson (2024) found that storytelling within organizations can either facilitate learning by enhancing information exchange and sensemaking or hinder learning by undermining trust in management. Storytelling, therefore, becomes a powerful tool for navigating tensions, shaping organizational members’ understanding of change and its implications (Buchanan and Dawson, 2007; Gabriel, 2000; Polkinghorne, 1988).

One form of storytelling, known as sensegiving, is employed by managers to frame the crisis in ways that justify rapid changes or reinforce existing hierarchies (Drori and Ellis, 2011; Kapucu and Van Wart, 2008). At the same time, the need for distributed and adaptive leadership opens space for alternative narratives, stories shared by frontline staff, patients, or departments that may be overlooked. However, these competing narratives often reveal deeper power struggles over whose interpretation of the crisis should guide organizational learning and resource allocation. If not addressed carefully, these power struggles can escalate into power games, ultimately eroding trust within the organization (Rosenbäck and Svensson, 2024). As Rosenbäck and Svensson (2024) highlight, trust is a critical element for learning, particularly during a crisis, and must be diligently safeguarded from the influence of power games and unanchored decisions.

This paper focuses on the phenomenon of power games as they unfolded during the COVID-19 pandemic, aiming to contribute to a deeper understanding of how these dynamics influence organizational learning, decision-making, and resilience in healthcare. While previous research has examined crisis management, sensemaking, and storytelling in isolation, this study bridges these perspectives by exploring the interplay between formal and informal structures, top-down sensegiving, and emergent power games. By analyzing experiences across two Swedish hospitals, the study not only illuminates how power dynamics shape learning and adaptation during crises but also identifies mechanisms through which informal leadership and distributed decision-making can mitigate or exacerbate these effects.

The structure of the paper is as follows: First, we review the relevant literature, followed by the methodological approach. The findings section outlines the empirical insights gained which are then discussed in relation to existing research. Finally, we offer conclusions and implications for practice and future studies.

## 2. Theoretical perspectives

Organizational change during crises is often marked by high uncertainty, urgent decision-making, and complex power dynamics. These dynamics shape decisions, influence whose voices are heard, and affect how adaptation occurs. In crisis situations, power struggles emerge both subtly and overtly, as different groups within the organization, negotiate meaning, assert control, and influence outcomes (Drori and Ellis, 2011). Power dynamics become deeply embedded in the processes through which individuals and groups attempt to manage the crisis and the emerging changes. This significantly affects how organizations respond to crises, how leaders and employees make sense of the situation, and how knowledge is shared or suppressed (Weick *et al*., 2005). Understanding the role of power games and the interplay between formal and informal structures is essential to comprehending organizational response and resilience during crises (Buchanan and Dawson, 2007).

### 2.1 Power games and the impact of sensemaking and sensegiving

As power games unfold, the negotiation of legitimacy becomes a central aspect of both sensemaking, which involves making sense of a situation, and sensegiving, which involves conveying the desired interpretation. Weick *et al*. (2005) argue that sensemaking is inherently social and political, with power struggles shaping which interpretations are accepted. Managers often use sensegiving to reinforce their narrative, maintain control, and justify decisions, sometimes marginalizing perspectives from lower-level staff. Through this process, managers not only create a coherent narrative but also influence behaviors and expectations across teams (Drori and Ellis, 2011). However, marginalizing alternative narratives can produce fragmented understanding, where some voices are amplified and others silenced (Balogun and Johnson, 2004; Reissner, 2006).

In crisis situations, sensegiving becomes a vital tool for maintaining organizational inertia and managing uncertainty. Leaders attempt to navigate competing interests and conflicting perspectives by strategically filtering and highlighting information to maintain stability, ensuring that the narrative they offer is embraced as the singular truth. This process becomes even more critical in times of crisis, where the sheer volume of contradictory messages can overwhelm decision-makers and undermine confidence in leadership (Drori and Ellis, 2011; Weick *et al*., 2005). Crisis management frameworks, such as the NATO model, outline top-down communication channels (NATO, 2025). When formal models diverge from the need in practice, informal methods often emerge, bypassing official channels and creating disjointed information flows. Over time, these informal networks can undermine formal authority, adding confusion and distrust (Buchanan and Dawson, 2007).

### 2.2 The impact of power games on organizational effectiveness

Organizational effectiveness during a crisis depends on organizational resilience, decision-making, and learning (Rosenbäck and Svensson, 2024). Resilience is shaped by how power struggles are navigated. Power games can either support or hinder adaptive responses, depending on leaders’ ability to balance competing interests and negotiate power struggles (Drori and Ellis, 2011).

Competing narratives within organizations reflect differences in experience and hierarchical position (Buchanan, 2003). Power struggles emerge in decision-making processes, with different groups using narratives to assert legitimacy, challenge authority, or influence the direction of change. When managers strategically use sensegiving, it can reinforce existing power structures rather than promote collective learning (Drori and Ellis, 2011). In healthcare settings, this dynamic may create confusion and frustration, particularly when decisions are not clearly communicated or fail to involve all key stakeholders, including employees and patients (Reissner, 2006).

The process of organizational learning is deeply influenced by power dynamics, especially in times of crisis (Buchanan, 2003). During crises, negotiating power is critical to maintaining trust in organizations. Poorly managed struggles can erode trust, undermining both organizational learning and resilience (Rosenbäck and Svensson, 2024). Moreover, Rosenbäck and Svensson (2024) suggest that resilience is not solely shaped by organizational learning but also by the ability to navigate the power struggles that influence decision-making and resource allocation during crises. The PDSA cycle (Deming, 1986) provides a framework for continuous learning, yet its effectiveness is shaped by the power relations within the organization. Power games intersect with the PDSA cycle, determining which stories are heard and whose interpretations are validated and if they will affect the continuing of the cycle (Rosenbäck, 2024).

Emergent informal processes may bypass official channels, create a disjointed flow of information, and make it difficult to implement cohesive strategies. Moreover, as informal methods become more dominant, they can undermine the authority of the formal leadership structure, contributing to confusion and distrust among stakeholders (Buchanan and Dawson, 2007). In this way, the gap between formal crisis management frameworks and actual practices on the ground becomes a breeding ground for power games, where those controlling informal networks can exert significant influence over organizational outcomes. This gap between formal frameworks and actual practice can hinder effective decision-making and leadership, particularly in high-pressure situations that demand flexibility and rapid decision-making (Kapucu and Van Wart, 2008).

When struggles are poorly managed, responses can fragment, with factions pursuing conflicting goals based on their interpretations. In such environments, power struggles can escalate into fully-fledged power games, creating deeper conflicts and further eroding trust and cohesion (Kapucu and Van Wart, 2008).

### 2.3 Overcoming power games

An approach to mitigate destructive power games is lateral leadership (Kühl *et al*., 2005), which focuses on creating shared understanding, shifting power dynamics, and building trust. Lateral leadership brings hidden power struggles to the surface by uncovering assumptions, conflicting interests, and communication patterns that can polarize groups. By inviting new actors into dialogue and reframing relationships, it fosters collaboration and unlocks new problem-solving potential.

Kühl *et al*. (2005) emphasize that power is inherently asymmetrical yet reciprocal since leaders depend on followers’ compliance, and those with limited formal power have stakes in maintaining relationships. Lateral leaders identify sources of influence, interests at play, and areas of uncertainty, creating common ground for collective action (Kühl *et al*., 2005).

Complementing this, Huy (2001) describes lateral leaders as those who manage tensions between continuity and change through trust, communication, and alignment with stakeholders’ perceptions. Lateral leadership does not eliminate power games; instead, it transforms them into constructive forces that overcome organizational blockages. Approached with openness and shared understanding, power games can enable coordinated action and drive organizational change (Kühl *et al*., 2005; Moore *et al*., 2023).

## 3. Methodology

### 3.1 Research design

This study is based on a qualitative case study design involving two public hospitals in Sweden, with the aim of exploring how power games influence organizational learning and resilience during the COVID-19 pandemic. A qualitative approach is particularly suited for examining management practices and complex social dynamics within organizational contexts (Flyvbjerg, 2006). The comparative case design, allows for the identification of both common patterns and context-specific variations, enhancing the analytical richness and the potential for theoretical generalization.

The selection of the two case hospitals was purposive and strategically grounded in both practical and analytical considerations. The cases represent contrasting contexts in terms of hospital size, organizational structure, regional governance, managerial experience, and the timing and severity of the pandemic’s impact. Importantly, the two hospitals are situated in different types of regions: one in a densely populated metropolitan area, and the other in a sparsely populated region with a larger geographical spread and longer travel distances between care units. These regional differences bring distinct organizational challenges and adaptive demands, particularly in terms of coordination, communication, and resource distribution.

In addition to these contextual contrasts, both hospitals were accessible to the researchers through established collaborations and trust-based relationships. This facilitated in-depth access to key stakeholders at multiple organizational levels, including senior management and frontline staff. The strength of these relationships also contributed to the credibility and openness of the data collection process, as participants were more willing to share their experiences candidly.

By combining variation in regional and organizational context with strong research access, the case selection enhances both the internal validity and the transferability of findings. The study thus provides a nuanced understanding of how organizational resilience is shaped under different conditions, while also contributing to broader theoretical insights into learning and adaptation during systemic disruption. Case studies are particularly valuable for such purposes, as they allow for detailed, context-sensitive analysis of real-world practices (Annamalah *et al*., 2025).

### 3.2 Practical context

Case A is a medium-sized emergency hospital located in an urban region with approximately 1,300 employees, where the virus spread early. At the onset of the pandemic, the region where the hospital is located was in the process of implementing NATO-based contingency plans. The region has several other hospitals, including larger university hospitals with extensive ICU capacity and smaller emergency hospitals.

**Figure 1:**
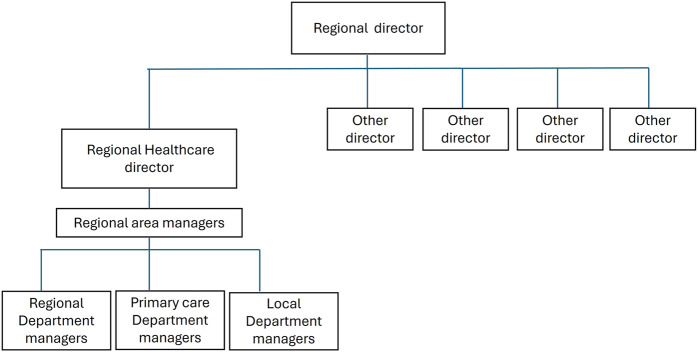
Regional organization where Case B is situated. Source: Authors’ own work

Case B is a large university hospital with about 6,000 employees, located in a region with two smaller emergency hospitals nearby. The virus spread later in this region, providing more time for preparation. Prior to the pandemic, this region had appointed a new Regional Healthcare Director (Healthcare director) who reorganized the organization by introducing area managers responsible for both regional departments, local departments and primary care (Figure 1).

**Figure 2:**
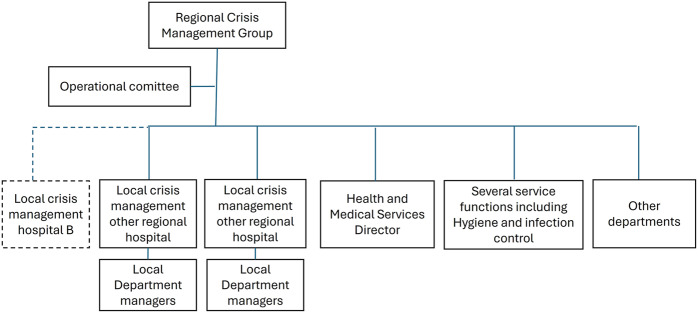
Regional crisis organization where Case B is situated. Source: Authors’ own work

The contingency plan in this region had not been updated to match the new organizational structure, and it still relied on local crisis management (LCM) (Table 1 for acronyms) at each hospital (Figure 2). The difference in organization meant that hospital B did not initiate a LCM. The normal healthcare organization operated parallel to the operational committee.

**Table 1:** Acronyms used for different roles and departments at the hospitals

Acronym	Description of role
CEO	Chief executive officer
DM	Department Manager
EC	Emergency Clinic
ICU	Intensive Care Unit
IC	Infectious diseases Clinic
IMC	Internal Medicine Clinic
LCM	Local Crisis Management
RCM	Regional Crisis Management
RHM	Regional Healthcare Management

**Source(s):** Authors’ own work

These organizational differences position the hospitals as “critical cases,” providing an opportunity for context-sensitive analysis (Flyvbjerg, 2006).

### 3.3 Data collection

Semi-structured interviews, used to encourage the participants to talk freely (Gioia *et al*., 2012), were conducted with 41 participants across both hospitals. Case A contributed 27 interviews, including the CEO, chief medical officer, and managers from operations, HR, education, communication, inpatient and outpatient care, ICU, and logistics. In Case B, 14 interviews were conducted, with both higher regional roles, such as Chief of regional crisis management (Chief RCM), Regional crisis coordinator, Regional chief physician, infection control officers, the regional, healthcare director and department managers (DMs) at the hospital. The interviews were conducted via video conferencing and lasted approximately one hour each. All interviews were recorded and transcribed verbatim. Only the interviews interesting for this paper are used here.

Based on the concept of information power proposed by Malterud *et al*. (2016), a sample of 41 participants was considered sufficient for the purpose of this study. According to this framework, the adequacy of the sample size in qualitative research is determined not by numbers alone, but by the richness and relevance of the information each participant contributes in relation to the study aim. Given the specificity of the research question, the sample’s homogeneity, and the strong quality of dialogue during interviews, it was assessed that the sample held sufficient information power to support meaningful analysis and draw valid conclusions.

The interviews were complemented by the secondary sources, which helped to contextualize the interview narratives and provided broader perspectives on the institutional and societal dynamics at play. This multi-source approach enhanced triangulation and contributed to a rich understanding of the organizational contexts, at the same time mitigating the possibility of subjectivity (Annamalah *et al*., 2025; Walsham, 2006). Triangulating the sources allowed to cross-validate findings, identify patterns, and detect possible contradictions, thereby enhancing the analytical rigor. This approach also helped to mitigate the risk of producing a distorted or overly narrow understanding of the studied phenomenon. By integrating first-hand accounts with external, independently produced material, the study was better equipped to capture the power dynamics within the healthcare sector during the crisis (Cauchick-Miguel *et al*., 2024).

### 3.4 Data analysis

Thematic deductive analysis was conducted using NVivo12. The analysis applied theoretical concepts from organizational learning (Argyris and Schön, 1997) and the learning cycle (Boin and ‘t Hart, 2006), as well as the concepts of power games and sensegiving (Gioia and Chittipeddi, 1991; Weick *et al*., 2005) and power and leadership dynamics (Drori and Ellis, 2011; Gioia and Chittipeddi, 1991). Based on this deductive analysis, three critical events were selected. The events were selected due to evident power struggles and differences in management levels, highlighting various types of organizational challenges. These challenges involved either ensuring that practical work could function or complying with crisis regulations.

The events are described as narratives, following a temporal flow of events and happenings (Polkinghorne, 1995). These narratives depict people’s situated actions and their perceptions thematically, highlighting purposeful engagement within the organizations. They also synthesize diverse events, actions, and experiences into coherent accounts. The focus in the results is on what people discussed in relation to the theoretical concepts, which is presented as stories within the narratives (Czarniawska, 1997). In this way, the narratives provide explanations of power games and their role in organizational learning and resilience, thereby addressing the aim of the study. However, the aim has not been to generalize findings from the cases to other hospital organizations.

### 3.5 Ethical considerations

Participants were informed both orally and in writing about the purpose of the study, the voluntary nature of participation, and their right to withdraw at any time. Written consent was obtained prior to each interview. Interviews were anonymized during transcription, and all data were stored securely to ensure confidentiality.

The study adheres to the ethical principles outlined in the Declaration of Helsinki (World Medical Association, 2013). As the research did not involve sensitive personal data, ethical approval was not required according to Swedish legislation. Ethical responsibility was maintained throughout, from data collection to the dissemination of findings.

## 4. Result

The results reveal that several interviewees experienced power games, ranging from smaller, individual power struggles, such as attempts to secure more equipment or avoid assignments in departments where one feels insecure, to more significant conflicts. To describe the power games during the pandemic, three distinct episodes are thoroughly examined, supported by quotes from the managers involved. These episodes are:

1. Episode 1: Power game over who should manage the prioritization of healthcare between the management of Hospital A and the regional crisis management (RCM) and political management.
2. Episode 2: Power game over who should manage the healthcare organization between the RHM and the RCM for Hospital B.
3. Episode 3: Power game over the practical needs and how to address them between the DMs at Hospital B and the RHM.

In Episode 1, the interviews are based solely on one party’s perspective, the hospital, although informal contacts, media, and other documents provided input from the regional perspective. In contrast, Episodes 2 and 3 draw on perspectives from both sides of the power struggles. These three power games were primarily triggered during the first wave of the pandemic, when the crisis was in its acute phase, filled with uncertainty.

### 4.1 Episode 1

Hospital A studied in this region were among the first to grasp the full severity of the pandemic, largely through the international network between infection specialists and intensive care physicians. However, the region’s crisis management system struggled to provide support. The regional crisis plan had been designed with short-term accidents in mind, where information typically flowed directly from the rescue leader. In contrast, during the pandemic, the crisis management team received second-hand information and found themselves unable to make timely, informed decisions. When they eventually initiated the crisis command, they overwhelmed the hospitals with unnecessary, detail-oriented questions about issues that had already been resolved. By the time they gathered the needed information, it was often too late to act effectively. As the *DM, of the intensive care unit (ICU)* explained:

The contact with the RCM broke down in the beginning …. and the hospitals solved the task …. The crisis management came more to see how things had turned out and blessed it afterward.

Also, the *Chief Medical Officer* described the frustration that arose because of the number of questions and decisions from regional level:

I spent a lot of time handling all these decisions that came through (from the RCM) – we had a functional inbox that could have driven anyone crazy trying to monitor it and make sure to address these decisions and communicate them …

Early in the pandemic, line organizations in the hospitals decided to collaborate directly with each other, by passing the RCM. As a result, the RCM was sidelined, which, in turn, contributed to delayed and often misguided decisions.

*Chief Executive Officer (CEO)* reflected on the evolving power game between RCM and the regular hospital management during the crisis, and the following development of a production coordination group:

We were under RCM throughout the first wave. Then, a rather intense discussion broke out, and a bit of power struggle between the RCM and the regular hospital lines, more specifically, the CEO group called the coordination group emerged …. But RCM has been part of this production coordination group … but in terms of power, it’s been weakened ….

This lack of alignment and clear communication caused friction between the hospital’s operations and the regional directives. *Head of Bed Management* further emphasized how local realities influenced their decisions:

I probably based my plans more on what was actually happening in reality. If they said, “You need 36 COVID beds,” and we had 42 patients in the flow, then I have 42 beds.

As the pandemic progressed, political interference became more pronounced, with a particular focus on establishing an external ICU. This proposal was met with resistance from the physicians, who saw the initiative as misguided. *DM of the ICU* described the situation:

The politicians wanted to set up a single emergency department for all the region … And the initiative to start everything came through a private healthcare provider. The Princess was made to go around there, while the politicians saw great photo opportunities, instead of having to approach the healthcare staff, who might throw tomatoes at them.

As the pandemic progressed, political interference became more evident, particularly when the focus shifted to managing the health care queues, which ultimately reduced the potential for effective cooperation between hospitals to achieve the most efficient production. The *DM of the ICU* reflected on how political decisions began to influence hospital dynamics:

The politicians said: “well, dear CEO, if you make sure you don’t take in too many COVID patients in your flow – but do a little bit of other things on the side so there isn’t so much media coverage about the queues – then you can get a gold star”

The *DM of the ICU* continued, highlighting the consequences of this approach:

And then the ICU chiefs sit down and start fighting with each other, and you’ve implemented “divide and conquer” all the way down to the healthcare floor, and it creates a completely different dynamic than it was in the first wave, where it was the exact opposite.

This shift in political influence had a significant impact on hospital decision-making.

*The CEO* also touched on the uncertainty surrounding the university hospital’s plans, which further complicated matters:

Then, what has become a challenge with the discussions around the changes is how to assess what the university hospital plans to do. It’s also become a bit of a game, you see. Because, how big will their capacity be? We, … must rely on the big player, and must consider how they plan to act, so to speak.

The political inference on the operations was disturbing the cooperation between the hospitals in the region.

### 4.2 Episode 2

An outdated crisis plan at hospital B made the crisis management unclear. A power game between the Chief of RCM, which wanted to manage strictly according to the plan, and the Healthcare Director, who wanted to lead healthcare “as usual,” led to different interpretations of responsibilities during the pandemic. The RCM argued that there should be an LCM at each of the three hospitals in the region, while the smaller hospitals did, the largest hospital in the region was still managed by the Healthcare Director.

The *Chief RCM* began by providing a very detailed explanation of the importance of crisis management and what it entails:

I believe that those working with crisis preparedness are the ones who perhaps see the entire societal picture of such a crisis. Because they are constantly in collaboration, at the regional level, with the county administrative board and all the municipalities … Healthcare is very focused on its task … But such a crisis is truly a societal disruption, and it requires a lot of collaboration at all levels … It includes a crisis management organization with various roles that are established and should be trained and exercised.

However, soon after, complaints were pronounced by *Chief RCM* regarding the Healthcare Director who did not follow the crisis management plan and did not start up the LCM at Hospital B:

We were too few who were trained and exercised. And that, in turn, led to the methodology not working very well. For example, the LCM was never activated in the largest municipality, where we had the biggest hospital … it was the Healthcare Director, who made the decision not to open it … And that led to confusion.

The *Healthcare Director*, however, had a different perspective on the events during the first wave:

We, from the healthcare leadership, have tried to influence it by emphasizing … that the care should be managed as similarly as possible to normal conditions, even during a pandemic … You might be talking to the Chief of RCM, and they have a slightly different view on this. I think they believe it’s working well and that their organization is functioning well. And I perceive that it works, because we manage the care without arguing about how the crisis management organization should be structured … Well, we were like the subcommittee of RCM, you could say. I mean, I went into it (RCM) and reported, and there was service and HR and all of them … But I don’t want to make a big deal out of it. Because it’s so sensitive, even though it’s a bit odd. But you have to choose your battles.

At the start of the pandemic, many found the organization confusing and disorganized.

The *Regional Chief Hygiene Physician*, for example, described the situation:

The regional director sat in that RCM team … where I was and acted. And then the Healthcare Director with team, the regional healthcare management (RHM), in a way, ran in parallel. And there, they didn’t really follow the pandemic plan; it was more … in the worst panic.

Instead of establishing separate LCM at each location, the RHM opted for a centralized approach under the RHM. The *Area Manager who later became the Chief LCM* saw this decision as somewhat fortuitous:

… So, instead of setting up LCM in each location, we had the RHM … And it turned out to be a stroke of luck, because then we were sitting together with the smaller hospitals. We were in the same group, so we could solve everything without going outside or escalating to the regional staff.

The formal discussions within the RCM and RHM were slow and took valuable time away from focusing on the urgent task of managing pandemic patients. The region where Hospital B is located was later affected by the pandemic waves, and fortunately, it experienced a lower spread of infection. While there were no major disasters, the decision-making process during the first wave was marked by significant confusion and a lack of clarity.

This disarray was reflected in the confusion of the *DM of the infectious diseases clinic (IC)*, as they struggled to determine who was making key decisions, who expressed this uncertainty:

It was the RHM who made the decision. Let’s see, if it … yes, it was … if it existed then. Yes, it probably existed. At least … we’ll say it was [laughs] … yes, it was RHM or RCM … It’s been a bit complex regarding whether the area managers and this operations management … It’s clear that sometimes it’s been a bit unclear. What decisions are made in the crisis management team? And then the other area managers, those who aren’t allowed to participate, can they also influence, or is there another forum on the side where they also want to influence?

This confusion within the decision-making process highlights the challenges faced by the region in managing the crisis effectively, particularly in the early stages when clear lines of authority and communication were not firmly established.

Despite the apparent success in addressing the clinical tasks, the *Area Manager (Chief LCM)* acknowledged the challenges of managing such a fluid and fragmented situation:

It was messy for us too … I could sit on three chairs sometimes, as a representative for the largest hospital, for my own organization, and as part of the RHM. So, there were some difficulties around the decisions … But it is incredibly difficult to trace things back.

In the second wave, the LCM at the largest hospital was established. This shift provided a clearer structure for the crisis organization, and the decision-making processes became faster and more efficiently documented. As the *Chief RCM* explained:

And then, from the fall, when the second wave came, we kicked off the crisis management organization as it was meant to function

This improved organization and communication made things run much more smoothly. For the *Area Manager (Chief LCM)*, the second wave brought more clarity to their responsibilities:

In the fall, I have been the chief of the LCM together with another area manager. So, we’ve been responsible for the largest hospital … And then, we’ve had a much clearer assignment

While the changes introduced during the second wave brought more clarity and structure, the *Healthcare Director* still felt that the organization could be improved and noted:

And now, in this second wave, we got a better organization that was more adapted to handle a pandemic … So now we have some kind of hybrid … As I said, when we restarted this fall, we said we can’t have the same organization as last time … then they (the RCM team) agreed to call it the regional COVID crisis healthcare team, meaning we want a healthcare leadership for COVID. And there, service and all the facilities join …. We have our own meetings, and we’re the ones managing the care …. But I think – It doesn’t fully follow the regulations.

Despite these improvements, the structural changes were not without tension. The regional director, who had been largely invisible throughout the pandemic, was replaced. Later, following the course of the pandemic, the Healthcare Director was also replaced, marking a significant shift in management after the crisis.

### 4.3 Episode 3

The third episode centers on a power struggle between the RHM and the DMs at Hospital B. This conflict arose from the RHM’s delays, lack of clarity, and at times, complete absence of decisions on how to increase hospital capacity and manage the pandemic. Hospital B also lacked direct communication with the RCM via LCM and was excluded from key decisions when the RHM failed to pass on relevant information. tThe DMs at Hospital B became increasingly frustrated with the lack of support from the RHM. As key decisions required to manage the crisis were overlooked or delayed, the DMs felt they were left to handle the escalating pressure alone.

*DM of the Emergency Clinic (EC)* shared this sentiment, noting how the RHM initially failed to recognize the scale and duration of the crisis:

Initially, I don’t think the RHM understood either, that this would go on for a long time and would put the entire hospital under strain … which led to decisions that needed to be made not being taken.

*DM of the IC* also felt the pressure of managing the pandemic’s immediate demands while being sidelined by RHM’s focus on future waves rather than present needs:

Our view was that they (RHM) focused very much on the upcoming wave and how we would staff and manage capacity, and so on … but we felt that we were missing the part of “what needs to be done right now?” This is urgent.

The *DM of the Internal Medicine Clinic (IMC)* echoed the lack of decisive leadership and added problems of documentation during the early stages of the pandemic:

Very few decisions were made … The first time, there wasn’t even any documentation. There was no traceability.

The same DM expressed astonishment at the organization’s lack of preparedness to handle such a large-scale crisis along with severe failures of the management style from the RHM.

I find it completely fascinating how, as an organization, we were so poorly equipped. I mean, organizationally … I think the leadership has been absolutely terrible … The organization is very top-down.

This outcry reflects the frustration and exhaustion of the DMs as they navigated not only the external pressures of the pandemic but also the internal challenges of a RHM that were slow to adapt to the realities of the crisis. The lack of timely and practical decisions left the departments relying on their own initiatives to maintain care and manage the surge of patients.

A group of the DMs which were most involved in the care of COVID-19 patients at the hospital initiated their own local decision-making group, which included the *DM of the IC*, along with colleagues from internal medicine, geriatrics, and emergency care.

… I, along with the department heads of medicine, geriatrics, and the emergency department, began meeting with our respective medical managers and … yes. So, it was really us …

This created an informal leadership structure, with the DMs acting together to manage the crisis, sidestepping the RHM as the *DM of the IC* expressed it:

It becomes informal leadership, so to speak. And a network … And it was the same spirit among everyone … that here we must contribute … We are all in the same boat.

The *Area Manager (Chief LCM)* did know about the grouping and that they took lot of initiatives:

Yes, we were aware of the groupings. But the management of them and what was discussed in detail, and who the main questioners were unclear … Questions came up from this group … And it was good that action was taken quickly based on the here-and-now situation.

*DM of the EC* recalled being part of this group but said that the group had limited mandate:

I was part of that group. But we were a group with limited mandate, meaning we only had authority over our own areas …... But it still went well, we’re still satisfied with what we managed to accomplish [laughs] with the resources we had.

While the DMs stepped forward, their initiative faced challenges. Despite their clear actions and good intentions, the DMs struggled to gain acceptance from the formal RHM. As a result, the DMs were forced to operate in the background, working outside the official channels.

The *DM of the IC* described the situation of tension:

But at certain points, one might have thought that here are some people stepping forward and doing things without having been given the task or mandate … Yes. Yes, that we, in a way, took up too much space … We just wanted to do our best, and we realized that we had somewhat different focuses.

They clarified that their goal was never to challenge the leadership but to ensure that the hospital could continue to operate effectively during the pandemic. Reflecting on the discomfort of working outside the formal structures, the *DM of the IMC* added:

Then they (RHM) thought we had established our own group and were opposing the hospital leadership, which we had no intention of doing at all … We worked like some kind of guerrilla warfare … We received a lot of criticism for it from above and were told not to speak up loudly and all sorts of things. Horrible.

This quote captures the internal tension and the difficult position the department managers found themselves in, trying to act in the best interest of the hospital while being undermined by a lack of formal support and receiving criticism from above. The criticism from RHM went so far so the *DM of the IMC*, who tried to overcome the situation by openness were dismissed:

Let’s put it this way … I’ll say what I think, because I believe it’s important, and I think we should have that openness. And it … it didn’t go well … I was asked to step down …

The *DM of the IMC* expressed the strong need for formal structures, emphasizing that clearer support was crucial to their continued effectiveness.

And we said next time that we refuse to sit as some group in the basement, this needs to be formalized.

Following a review by the Swedish Civil Contingencies Agency (MSB) and a restructuring of the organization, the LCM team was officially established at the hospital B with one of the Area managers as Chief LCM. The DM group, who had previously acted independently, were integrated into this formal structure. This transition helped formalize the group’s role, making their work more recognized and appreciated.

*Area Manager (Chief LCM)* described the process of forming a more formal group:

This group (the DM’s group) and the LCM have now merged. I think we have a great dialogue now thanks to our weekly check-ins with the departments … I don’t experience the same demand from the departments for information or “you’re unclear … ”

Also, the *DM of the IMC* appreciated the report and the agreement that the management did not follow the intentions.

It felt really good when the MSB report came, because it highlighted exactly this, that we weren’t following the guidelines that exist in a crisis situation …

Theses statement highlights the improvement in communication and coordination after the formalization of the crisis management structure, where roles were clearly defined, and departments felt more engaged and informed. While the DMs appreciated the recognition, there was still an underlying sense of discomfort, as the credit came only after the situation had unfolded, and they felt that their actions were not fully supported at the time, and the DM of the IMC did eventually resign from the management.

## 5. Discussion

This study highlights the significant role that power games and leadership dynamics played in shaping the organizational response to the COVID-19 pandemic within healthcare settings. The findings show that leadership during crises, particularly in healthcare organizations, is rarely a straightforward process. Instead, it is marked by complex interactions by sensegiving and power games between formal and informal power structures, which significantly impact the decision-making and overall effectiveness of the crisis management response in line with Drori and Ellis (2011) and Rosenbäck and Svensson (2024).

### 5.1 Top-down sensegiving effect on hospital management

A key finding of this study was the pervasive power games that emerged between crisis management according to formal plans and the hospitals’ usual management practices. Chiefs of RCM in both regions used sensegiving to provide directions aligned with NATO crisis management plans (NATO, 2025). However, when sensegiving was employed unilaterally by higher management, it risked marginalizing alternative narratives, particularly those from frontline staff, and thereby limited inclusivity and organizational learning (Drori and Ellis, 2011). This top-down approach generated frustration and confusion, as staff felt their on-the-ground insights were disregarded in favor of directives that were not grounded in the realities of the pandemic, consistent with previous research (Kapucu and Van Wart, 2008; Rosenbäck and Svensson, 2024). Communication disconnects further amplified power struggles between hierarchical levels and contributed to erosion of trust, as delayed and misaligned decisions fostered frustration and disillusionment among staff (Rosenbäck and Svensson, 2024).

As a result, the CEO of Hospital A addressed issues directly with other hospital managers rather than waiting for the RCM. Similar dynamics were observed in Hospital B, highlighting the generality of these findings. In Hospital B, the RHM established a decision-making structure parallel to the RCM operating committee. Buchanan and Dawson (2007) noted that, in crisis situations, informal working methods often emerged alongside formal structures, particularly when the latter could not adapt rapidly. While effective in the short term, this hybrid leadership created long-term challenges for organizational cohesion and clarity in decision-making. The comparative observations from both hospitals therefore reinforce the significance of top-down sensegiving on hospital management.Ambiguity in leadership roles, especially during the early stages of the pandemic, led to missed opportunities for effective collaboration. As Weick *et al*. (2005) noted, organizations had to be able to adjust quickly to changing circumstances, and rigid leadership structures impeded this adaptability. Similarly, Boin *et al*. (2016) argue that it is preferable to have a “plan for planning” rather than adhering strictly to a predetermined course of action.

### 5.2 Consequences of missing leadership support

Another key finding was that the absence of clear decision-making from top management levels contributed to frustration among second-line managers. Delays in key decisions and the exclusion of relevant information from the RHM left the DMs of Hospital B to fend for themselves, which led to the emergence of informal leadership structures that ultimately took charge of addressing immediate challenges. Their actions were driven by the urgency to meet immediate operational needs. Characterized by rapid, autonomous decision-making, these networks enabled hospital departments to continue functioning despite the absence of strong central direction. This dynamic reflected the tension between top-down decision-making and the need for more agile, localized leadership in times of crisis (Kapucu and Van Wart, 2008).

The DMs’ actions at Hospital B, while not officially sanctioned, created tensions with the RHM. The managers’ independent decision-making was perceived as a challenge to formal authority, which generated accusations of disloyalty and friction between groups, consistent with earlier research (Kapucu and Van Wart, 2008). These power struggles consumed valuable time and energy that could otherwise have been directed toward managing the crisis. The comparative study shows that trustful leadership is critically important for mitigating the consequences of missing leadership support.

### 5.3 Strategies to mitigate power games

To address the communication gaps and power games described above, the following strategies were implemented. During the COVID-19 crisis, the CEO group in the region including Hospital A began coordinating decisions based on information collected from each hospital. To reduce mismatches with the formal crisis management plan and prevent conflicting directives from the RCM, the RCM was invited to participate in the CEO group. This improved communication and enhanced trust between the RCM and the hospitals, illustrating how lateral leadership can mitigate power struggles and support coordinated decision-making (Kühl *et al*., 2005; Huy, 2001). However, later political involvement in decision-making complicated collaboration and remained unresolved throughout the crisis with decreased trust for them.

In Hospital B, escalating power games prompted an external review, which reinforced the formal crisis management structure. LCM was established, and the DM group was integrated into the formal framework. This demonstrates how clear communication, and structured inclusion can help align formal and informal decision-making processes (Kühl *et al*., 2005; Moore *et al*., 2023). It also highlights the importance of elevating professionals to contribute to overarching decisions within a more agile governance structure (Kapucu and Van Wart, 2008). However the missed opportunity to involve the DMs in practical decision-making combined with the RHM’s mistrust, which culminated in the dismissal of one DM (later revoked), ultimately led to the loss of an engaged manager who resigned from the position after the pandemic. Despite these improvements, there were still unresolved issues, particularly regarding the role of RCM. The Healthcare Director, for instance, expressed frustration with the slow adaptation of the RCM structures and continued with the parallel function reflecting persistent top-down dynamics. This unresolved power game ultimately resulted in the dismissal of the Healthcare director after the pandemic, highlighting the long-term impact of unmanaged power games on organizational cohesion and leadership effectiveness.

The mitigation of power games differed between the two hospitals, but the findings suggest that forming inclusive decision-making groups with all relevant stakeholders is a promising approach for future crises.

### 5.4 Power games and organizational learning

This study reveals the significant role of organizational learning during times of crisis in line with Argyris and Schön (1997). Learning was facilitated through both formal and informal channels. The pandemic presented a steep learning curve for all involved, with DMs quickly developing new processes for managing patients and safeguarding staff. However, as Rosenbäck and Svensson (2024) argue, learning is not always a straightforward process and can be hindered by power games. The decisions made by the DM group, but also the RHM, were often not documented and saved, leading to a lack of traceability and continuity in learning to organizational knowledge (Rosenbäck, 2024). This is a critical point, as organizational learning requires both clear documentation and the ability to adapt based on experience (Gherardi and Nicolini, 1998). The introduction of better documentation and clearer organizational structures during the second wave of the pandemic improved communication and decision-making, enabling the hospitals to respond more effectively to the demand. This study illustrates that the way power is exercised during crises directly influences the ability of the organization to capture and institutionalize learning from experience.

Evidence of organizational learning (Boin and ‘t Hart, 2006) and deliberate experimentation through PDSA cycles (Deming, 1986) emerged in this study. Sensing was frequently enacted through sensegiving (Gioia and Chittipeddi, 1991; Weick *et al*., 2005) to align the organization around a common course of action. While such sensegiving supported coordination, insufficient bottom-up sensing from frontline staff reduced agility and increased the risk of misdirected action. At the same time, managers and employees engaged in continuous experimentation and adjustments, generating new solutions and decisions. These iterative PDSA processes together with sensible sensegiving enabled the organizations to adapt and ultimately withstand the crisis (Rosenbäck and Svensson, 2024).

The comparative observations across Hospital A and Hospital B indicate that similar dynamics occurred in both contexts, strengthening the generalizability of the findings. However, the study also illustrates that the effectiveness of organizational learning is contingent on how power is exercised: when power games limit documentation, communication, or bottom-up input, the organization risks losing critical knowledge and reducing its ability to respond to future crises. Thus, managing power dynamics and ensuring inclusive participation are essential for translating learning into sustained organizational capability.

## 6. Conclusions

This study highlights the critical role of power dynamics and leadership structures in shaping healthcare organizations’ responses to the COVID-19 pandemic. Management during crises is rarely straightforward; it is characterized by complex interactions between formal and informal structures, sensegiving, and emergent power games.

Top-down sensegiving, when applied without understanding operational realities, reinforces existing power structures and marginalized frontline perspectives. Inability to align directives with on-the-ground needs contributes to mistrust, frustration, and decreases cohesion. These findings underscore that effective crisis leadership requires balancing centralized decision-making with decentralized, adaptive responses that incorporate frontline insights.

Informal leadership emerges as a crucial mechanism for addressing immediate operational challenges, particularly when higher management fails to provide clear and timely guidance. While effective in the short term, these informal structures sometimes create tensions with formal leadership, leading to fragmented decision-making and communication breakdowns. Integration of informal groups to formal crisis management when recognizing and structuring informal leadership can strengthen organizational response.

Organizational learning is deeply affected by these dynamics. Decisions made informally are often undocumented, limiting traceability and continuity of learning. Improved documentation and clearer structures facilitate better communication, coordination, and knowledge capture, demonstrating that the way power is exercised directly influences an organization’s ability to learn from experience.

Overall, this study emphasizes that trust, communication, and adaptive leadership are essential for effective crisis management. Healthcare organizations should prioritize flexible structures that integrate formal and informal leadership, encourage inclusive narratives, and enable decentralized decision-making. By doing so, organizations can enhance resilience, support continuous learning, and respond more effectively to future crises.

### 6.1 Practical implications

Based on the findings, the following considerations can help healthcare organizations avoid power struggles and strengthen crisis management in future pandemics:

1. *Use sensegiving with listening:* Sensegiving is valuable, but it should be grounded in input from frontline professionals before decisions are communicated.
2. *Adopt a “plan to plan” approach:* Crisis plans should emphasize adaptability, allowing management to adjust to emerging conditions rather than rigidly following a pre-set script.
3. *Include hospital-level management:* Actively involve department and unit managers in decision-making groups to ensure that decision-making is informed by accurate, on-the-ground knowledge.
4. *Prioritize practicality over politics:* Focus on operationally feasible solutions rather than symbolic decisions aimed primarily at external stakeholders.
5. *Embrace service-oriented leadership:* Leaders at all levels should act as enablers, listening to what is needed and ensuring the necessary resources and support are delivered.
